# Sex Difference in the Associations among Hyperuricemia with New-Onset Chronic Kidney Disease in a Large Taiwanese Population Follow-Up Study

**DOI:** 10.3390/nu14183832

**Published:** 2022-09-16

**Authors:** Jui-Hsin Chen, Chun-Chi Tsai, Yi-Hsueh Liu, Pei-Yu Wu, Jiun-Chi Huang, Tung-Ling Chung, Ho-Ming Su, Szu-Chia Chen

**Affiliations:** 1 Department of Nursing, Kaohsiung Municipal Siaogang Hospital, Kaohsiung Medical University, Kaohsiung 812, Taiwan; 2 Department of Occupational Safety and Health, Kaohsiung Municipal Siaogang Hospital, Kaohsiung Medical University, Kaohsiung 812, Taiwan; 3 Division of Cardiology, Department of Internal Medicine, Kaohsiung Medical University Hospital, Kaohsiung 807, Taiwan; 4 Department of Internal Medicine, Kaohsiung Municipal Siaogang Hospital, Kaohsiung Medical University, Kaohsiung 812, Taiwan; 5 Division of Nephrology, Department of Internal Medicine, Kaohsiung Medical University Hospital, Kaohsiung Medical University, Kaohsiung 807, Taiwan; 6 Faculty of Medicine, College of Medicine, Kaohsiung Medical University, Kaohsiung 807, Taiwan; 7 Graduate Institute of Medicine, College of Medicine, Kaohsiung Medical University, Kaohsiung 807, Taiwan; 8 Division of Nephrology, Department of Internal Medicine, Kaohsiung Veterans General Hospital, Kaohsiung 813, Taiwan; 9 Research Center for Precision Environmental Medicine, Kaohsiung Medical University, Kaohsiung 807, Taiwan

**Keywords:** hyperuricemia, new-onset chronic kidney disease, sex difference, Taiwan Biobank, follow-up

## Abstract

The global prevalence and incidence of chronic kidney disease (CKD) continue to increase. Whether hyperuricemia is an independent risk factor for renal progression and whether there are sex differences in the relationships between serum uric acid (UA) and a decline in renal function are unclear. Therefore, in this longitudinal study, we aimed to explore these relationships in a large cohort of around 27,000 Taiwanese participants in the Taiwan Biobank (TWB), and also to identify serum UA cutoff levels in men and women to predict new-onset CKD. A total of 26,942 participants with a median 4 years of complete follow-up data were enrolled from the TWB. We excluded those with CKD (estimated glomerular filtration rate <60 mL/min/1.73 m^2^) at baseline (*n* = 297), and the remaining 26,645 participants (males: 9356; females: 17,289) were analyzed. The participants who developed CKD during follow-up were defined as having incident new-onset CKD, and those with a serum UA level >7 mg/dL in males and >6 mg/dL in females were classified as having hyperuricemia. After multivariable analysis, hyperuricemia (odds ratio [OR], 2.541; 95% confidence interval [CI], 1.970–3.276; *p* < 0.001) was significantly associated with new-onset CKD. Furthermore, in the male participants (*n* = 9356), hyperuricemia (OR, 1.989; 95% CI, 1.440–2.747; *p* < 0.001), and quartile 4 of UA (vs. quartile 1; OR, 2.279; 95% CI, 1.464–3.547; *p* < 0.001) were significantly associated with new-onset CKD, while in the female participants (*n* = 17,289), hyperuricemia (OR, 3.813; 95% CI, 2.500–5.815; *p* < 0.001), quartile 3 of UA (vs. quartile 1; OR, 3.741; 95% CI, 1.250–11.915; *p* = 0.018), and quartile 4 of UA (vs. quartile 1; OR, 12.114; 95% CI, 14.278–34.305; *p* < 0.001) were significantly associated with new-onset CKD. There were significant interactions between hyperuricemia and sex (*p* = 0.024), and quartiles of serum UA and sex (*p* = 0.010) on new-onset CKD. Hyperuricemia was associated with new-onset CKD in the enrolled participants, and the interactions between hyperuricemia and sex were statistically significant. Hyperuricemia was more strongly associated with new-onset CKD in the women than in the men.

## 1. Introduction

The global prevalence and incidence of both chronic kidney disease (CKD) and end-stage renal disease (ESRD) continue to increase, including in Taiwan which has the highest prevalence of ESRD worldwide [1,2]. In 2017, an estimated 11.1% (843.6 million) of the global population had CKD, indicating the scale of the public health issue worldwide [3]. ESRD is associated with high rates of cardiovascular morbidity and mortality [4], and a rapid deterioration in renal function has been associated with increased rates of complications [5]. Therefore, it is very important to be able to detect the factors associated with a deterioration in renal function as early as possible.

Hyperuricemia refers to the excess production of uric acid (UA), which can be caused by a purine-rich diet, and increased purine metabolism and degradation. It can also be caused by a decrease in UA excretion due to acute CKD, excess alcohol consumption, acidosis, hypothyroidism, hyperparathyroidism, the use of diuretic agents, and lead poisoning [6]. Hyperuricemia is common in patients with CKD, which may be explained by the decrease in UA excretion as renal function worsens and the associations between hyperuricemia and CKD risk factors [7]. Some epidemiological studies have demonstrated that hyperuricemia is an independent risk factor for adverse renal outcomes [8] and the onset and progression of CKD [9,10,11], whereas others have not [12]. Therefore, whether hyperuricemia is an independent risk factor for a decline in renal function is uncertain. In addition, whether it only reflects the combined effect of renal damage with comorbidities or whether it is a causative factor is also controversial.

Furthermore, sex differences in the level of serum UA have been reported, with males generally having higher levels than females [13]. However, few studies have evaluated sex differences in the relationships between serum UA and a deterioration in renal function. Therefore, in this longitudinal study, we aimed to explore these relationships in a large cohort of around 27,000 Taiwanese participants in the Taiwan Biobank (TWB), and also to identify serum UA cutoff levels in men and women to predict new-onset CKD.

## 2. Materials and Methods

### 2.1. TWB

The TWB was developed by the Ministry of Health and Welfare in Taiwan with the goal of preventing chronic diseases by promoting health considering the problem of an aging society. Data on medical, genetic and lifestyle factors of participants aged 30 to 70 years living in communities around Taiwan with no prior history of cancer are recorded in the TWB [14,15].

### 2.2. Laboratory, Demographic, and Medical Variables

All participants in the TWB were interviewed and receive physical examinations during which blood samples were obtained. In addition, data on body mass index (BMI) (calculated as weight/height^2^), age, sex, and medical history (hypertension and diabetes mellitus) are recorded. The following laboratory data were also obtained: triglycerides, hemoglobin, fasting glucose, total cholesterol, high- and low-density lipoprotein (HDL/LDL) cholesterol, serum UA, and estimated glomerular filtration rate [eGFR] (calculated using the 4-variable Modification of Diet in Renal Disease study equation [16]). Regular exercise was defined as exercising at least three times a week for at least 30 min each time [17].

Blood pressure (BP) was measured digitally by trained personnel. The patients were instructed not to smoke, exercise, or consume caffeine-containing drinks for at least 30 min before BP was measured. Systolic BP and diastolic BP were measured three times each after a 1–2-min interval between readings, and average values were used in the analysis.

### 2.3. Study Participants

**Figure 1:**
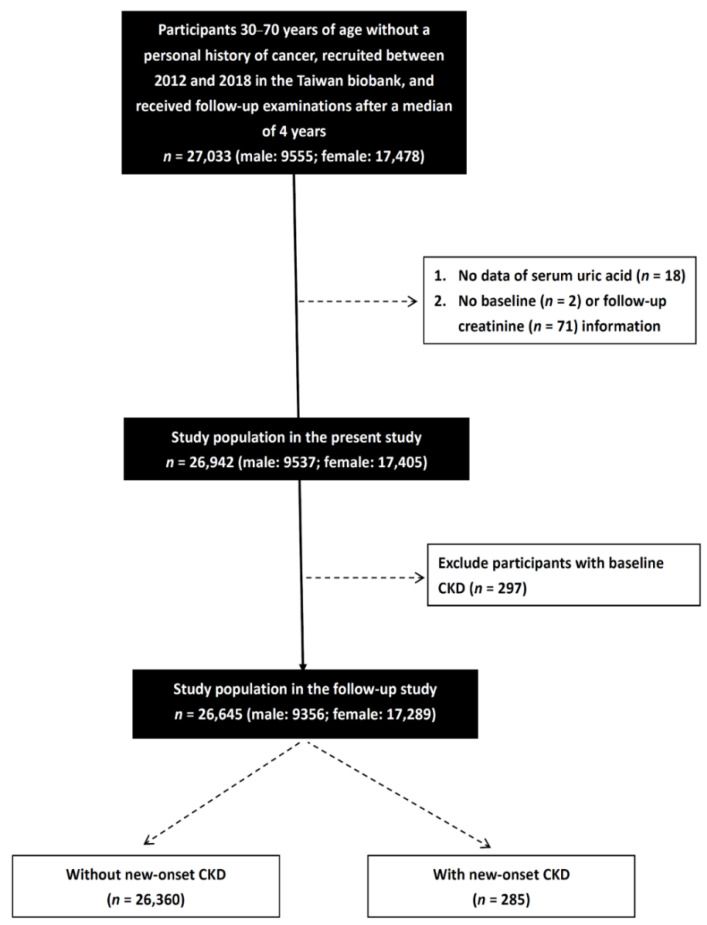
Flowchart of study population.

We identified 27,033 participants in the TWB with follow-up data for a median of 4 years and excluded those with no data on serum UA (*n* = 18), and those with no baseline (*n* = 2) or follow-up creatinine (*n* = 71) information. The remaining 26,942 participants (males: 9537; females: 17,405) were enrolled, all of whom provided written informed consent. We excluded those with CKD (eGFR < 60 mL/min/1.73 m^2^) at baseline (*n* = 297). Finally, a total of 26,645 participants (males: 9356; females: 17,289) were analyzed in this study (Figure 1).

### 2.4. Definition of New-Onset CKD

Participants with an eGFR ≥60 mL/min 1.73 m^2^ were considered not to have CKD. If these participants developed CKD (eGFR < 60 mL/min 1.73 m^2^) during follow-up, they were defined as having new-onset CKD.

### 2.5. Definition of Hyperuricemia

Participants with a serum UA level >7 mg/dL in males and >6.0 mg/dL in females were classified as having hyperuricemia [18].

### 2.6. Ethics Statement

The Institutional Review Board of Kaohsiung Medical University Hospital approved this study (KMUHIRB-E(I)-20210058). Ethical approval for the TWB was granted by the IRB on Biomedical Science Research, Academia Sinica, Taiwan and the Ethics and Governance Council of the TWB. In addition, the study was conducted according to the Declaration of Helsinki.

### 2.7. Statistical Analysis

Statistical analysis was conducted using SPSS (version 19 for Windows^®^, IBM Inc., Armonk, NY, USA). Data are presented as percentage or mean ± standard deviation. Differences between continuous variables were analyzed using the independent *t*-test, and differences between categorical variables were analyzed using the chi-square test. Multivariable logistic regression analysis was used to identify factors associated with new-onset CKD. Significant variables in univariable analysis were then analyzed using multivariable analysis. The quartiles of serum UA level were: ≤5.5, 5.5–6.4, 6.4–7.3 and >7.3 mg/dL in males, and ≤4.1, 4.1–4.8, 4.8–5.6 and >5.6 in females. Quartile 1 of serum UA was used as the reference as it had the lowest incidence rate. An interaction *p* in logistic analysis was defined as: model disease (y) = x1 + x2 + x1 × x2 + covariates; where x1 × x2 is the interaction term, y = new-onset CKD, x1 = sex, and x2 = hyperuricemia and quartiles of serum UA. The covariates were age, diabetes, hypertension, systolic and diastolic BPs, smoking and alcohol history, BMI, fasting glucose, hemoglobin, triglycerides and HDL-cholesterol in males, and age, diabetes, hypertension, systolic and diastolic BPs, smoking and alcohol history, BMI, fasting glucose, hemoglobin, triglycerides, HDL-cholesterol and menopause status in females. A *p* value of < 0.05 was considered significant.

## 3. Results

The mean age of the 26,645 enrolled participants was 51.1 ± 10.4 years and included 9356 males and 17,289 females. The participants were stratified into two groups according to the presence of new-onset CKD.

### 3.1. Comparison of Clinical Characteristics among the New-Onset CKD Groups

The new-onset CKD group were older, had more females, higher prevalence rates of hyperuricemia, diabetes mellitus and hypertension, and higher systolic BP, diastolic BP, smoking and alcohol history, BMI, serum UA, fasting glucose, hemoglobin, and triglycerides, and lower menstruation rate in females, HDL-cholesterol and eGFR than the without new-onset CKD group (Table 1).

### 3.2. Determinants of New-Onset CKD

The results of multivariable logistic regression analysis to identify the determinants of new-onset CKD are shown in Table 2. After adjusting for the significant variables in Table 1, older age, male sex, diabetes mellitus, hypertension, high systolic BP, alcohol history, high BMI, hyperuricemia (odds ratio [OR], 2.541; 95% confidence interval [CI], 1.970–3.276; *p* < 0.001), high fasting glucose, low hemoglobin, and high triglycerides were significantly associated with new-onset CKD.

### 3.3. Clinical Characteristics of the Study Participants Classified by the Presence of Different Sex and Hyperuricemia

Table 3 shows the clinical characteristics of the study participants classified by the presence of different sex and hyperuricemia. Compared to male participants without hyperurcemia, male participants with hyperuricemia were younger, lower prevalence of DM, higher prevalence of hypertension, higher systolic and diastolic BPs, higher prevalence of smoking and alcohol history, lower prevalence of regular exercise habit, higher BMI, higher UA, lower fasting glucose, higher hemoglobin, higher triglyceride, higher total cholesterol, lower HDL-cholesterol, higher LDL-cholesterol and lower eGFR. Moreover, compared to female participants without hyperurcemia, female participants with hyperuricemia were older, higher prevalence of DM, higher prevalence of hypertension, higher systolic and diastolic BPs, higher BMI, lower menstruation rate, higher UA, higher fasting glucose, higher hemoglobin, higher triglyceride, higher total cholesterol, lower HDL-cholesterol, higher LDL-cholesterol and lower eGFR.

Further, compared to male participants with hyperuricemia, female participants with hyperuricemia were older (*p* < 0.001), had higher prevalence of DM (*p* < 0.001), lower diastolic BP (*p* < 0.001), lower prevalence of smoking (*p* < 0.001) and alcohol history (*p* < 0.001), lower hemoglobin (*p* < 0.001), lower triglyceride (*p* < 0.001), higher total cholesterol (*p* < 0.001), higher HDL-cholesterol (*p* < 0.001), higher LDL-cholesterol (*p* < 0.001) and higher eGFR (*p* < 0.001).

### 3.4. Associations of Hyperuricemia and Quartiles of Serum UA with New-Onset CKD by Sex

The results of multivariable logistic regression analysis to identify associations of hyperuricemia and quartiles of UA with new-onset CKD by sex are shown in Table 4. In the male participants (*n* = 9356), after adjusting for age, diabetes, hypertension, systolic and diastolic BPs, smoking and alcohol history, BMI, fasting glucose, hemoglobin, triglycerides and HDL-cholesterol, hyperuricemia (OR, 1.989; 95% CI, 1.440–2.747; *p* < 0.001) and quartile 4 (Q4) of serum UA (vs. Q1; OR, 2.279; 95% CI, 1.464–3.547; *p* < 0.001) were significantly associated with new-onset CKD. In the female participants (*n* = 17,289), after adjusting for age, diabetes, hypertension, systolic and diastolic BPs, smoking and alcohol history, BMI, fasting glucose, hemoglobin, triglycerides, HDL-cholesterol and menopause status, hyperuricemia (OR, 3.813; 95% CI, 2.500–5.815; *p* < 0.001), Q3 of serum UA (vs. Q1; OR, 3.741; 95% CI, 1.250–11.915; *p* = 0.018) and Q4 of serum UA (vs. Q1; OR, 12.114; 95% CI, 14.278–34.305; *p* < 0.001) were significantly associated with new-onset CKD.

### 3.5. Interactions among Hyperuricemia, Quartiles of Serum UA and Sex on New-Onset CKD

There were significant interactions between hyperuricemia and sex (*p* = 0.024), and quartiles of serum UA and sex (*p* = 0.010) on new-onset CKD (Table 4).

## 4. Discussion

In this study, we explored differences between male and female participants in the association between hyperuricemia and new-onset CKD in a large Taiwanese cohort. The results showed that hyperuricemia, defined as a serum UA level >7 mg/dL in men and >6 mg/dL in women, was significantly associated with new-onset CKD. Furthermore, the interactions between hyperuricemia and sex were also statistically significant, and hyperuricemia was more strongly associated with new-onset CKD in the women than in the men. When analyzing serum UA levels by quartile, the odds of developing new-onset CKD was 2.28 in the men in Q4 (>7.3 mg/dL), and even higher in the women at 3.74 for those in Q3 (4.8–5.6 mg/dL) and 12.114 for those in Q4 (>5.6 mg/dL).

An important finding of this study is that hyperuricemia was associated with new-onset CKD in both the male and female participants. Several prior studies found that an elevated serum UA level was an independent predictor of the development of CKD. A cohort study of 6403 patients conducted by Iseki et al. revealed the relationship between elevated UA and new-onset CKD [9]. After controlling for other risk factors, a UA level of 8.0 mg/dL was associated with a 2.9-fold higher risk in men and a 10.4-fold higher risk in women of developing high serum creatinine [9]. Another prospective cohort study with 21,475 healthy volunteers who were followed prospectively for a median of 7 years reported that a modestly elevated UA level (7.0 to 8.9 mg/dL) was associated with an OR of 1.74 (95% CI 1.45 to 2.09) for new-onset CKD, while a markedly elevated serum UA level (≥9.0 mg/dL) was associated with an OR of 3.12 (95% CI 2.29 to 4.25) [10]. In addition, Weiner et al. examined pooled data from two community-based cohorts, the Atherosclerosis Risk in Communities (ARIC) Study and Cardiovascular Health Study (CHS), and found that an elevated serum UA level was a modest independent risk factor for a subsequent reduction in renal function (OR 1.07; 95% CI 1.01 to 1.14) [11]. However, some studies have not supported these findings. Chonchol et al. found no significant association between baseline UA level and new-onset CKD (OR 1.00; 95% CI 0.89 to 1.14), but a modest association between quintiles of UA level and the progression of kidney disease [19]. UA may be linked to new-onset CKD through several mechanisms: (1) UA may be an endogenous nephrotoxin; (2) an increased serum UA level may aggravate other risk factors associated with a decline in kidney function, particularly hypertension; and (3) UA may be a marker of other risk factors, including metabolic syndrome and diabetes [11]. Previous animal studies have demonstrated how UA impairs kidney function. In a rat model, hyperuricemia was shown to induce primary arteriolopathy of the preglomerular vasculature of the kidneys by stimulating vascular smooth muscle cells, thereby causing activation of the renin-angiotensin system and hypertension [20,21,22]. In addition, elevated UA has been shown to induce oxidative stress and endothelial dysfunction, leading to the development of systemic and glomerular hypertension [23]. An animal model study showed that hyperuricemia caused phenotypic changes in cultured tubular epithelial cells and renal tubules, leading to epithelial-to-mesenchymal transition of renal tubules and interstitial fibrosis [24]. Furthermore, intratubular precipitation of UA crystals has been shown to result in tubular obstruction, activation of local inflammation and interstitial fibrosis [25]. Clinically, many epidemiological studies have also demonstrated a link between an elevated UA level and hypertension [26,27,28], diabetes mellitus [29,30] and atherosclerosis [31,32], all of which may contribute to the development CKD.

Another important finding of this study is that the interactions between hyperuricemia and sex were also statistically significant, and hyperuricemia was more strongly associated with new-onset CKD in the females than in the males. Previous studies have investigated sex differences in the relationship between serum UA levels and the incidence of CKD, however, the results have been inconsistent. A meta-analysis of 13 studies found no significant differences in risk estimates between males and females with regards to the association between an elevated serum UA level and developing CKD [33]. Nevertheless, other recent research has shown that the trend of developing CKD in individuals with an elevated serum UA level differs between females and males. Weiner et al. reported that baseline serum UA level was associated with increased risk of new-onset CKD in females with an OR of 1.19 (95% CI 1.05 to 1.36) and a trend in men with an OR of 1.05 (95% CI 0.92 to 1.19) [11]. In addition, a longitudinal analysis of 138,511 middle-aged Japanese participants found that the association between serum UA level and the incidence of CKD may differ between men and woman. After adjusting for confounding factors, the hazard ratio (HR) for CKD incidence in women with a serum UA level ≥9.0 mg/dL was 3.20 compared to women with a serum UA level 4.0–4.9 mg/dL; while the HR was 3.74 in men with a serum UA level ≥11.0 mg/dL compared to men with a serum UA level 4.0–4.9 mg/dL [34]. Another cross-sectional study with 4242 elderly participants reported sex differences in the association between high serum UA and an increased risk of CKD [35]. The odds of CKD for those in the fourth quartile of serum UA levels were 6.05 (95% CI: 4.32–8.49) in males and 8.21 (95% CI: 5.37–12.54) in females compared to those in the first quartile of serum UA [35]. It is well known that there is a sex difference in UA metabolism, with women having lower serum UA levels than men due to the effect of estrogen [13,36], which can promote the excretion of UA [13] and inhibit xanthine oxidase to generate UA [37]. Nevertheless, the mechanism for the stronger association between hyperuricemia and new-onset CKD in the females in this study remains uncertain. There are several potential explanations. First, the balance of UA metabolism may be disrupted more in women than in men at the same level of serum UA. Women with hyperuricemia may have an unhealthier diet and lifestyle than men with hyperuricemia [34]. Second, increased serum UA is associated with other risk factors for developing CKD, including hypertension, diabetes, BMI and lifestyle, which may reduce the excess effect of an elevated UA level. Men may have other risk factors associated with serum UA level and the development of CKD to women. Finally, in our study, female participants with hyperuricemia were older, had higher DM prevalence, higher total cholesterol, and higher LDL-cholesterol, which might possibly contribute to new-onset CKD.

Another interesting finding is that the men in serum UA Q4 (>7.3 mg/dL) had an OR of 2.28 for new-onset CKD, compared to 3.74 in the women in Q3 (4.8–5.6 mg/dL), and 12.114 in the women in Q4 (>5.6 mg/dL). There is general consensus on initiating urate-lowering therapy in individuals with symptomatic hyperuricemia. However, the benefits of treating hyperuricemia in asymptomatic individuals is still controversial, and clinical data on the use of urate-lowering therapy to prevent the incidence and progression of CKD are conflicting. In 2012, the Kidney Disease Improving Global Outcomes Clinical Practice Guideline for the Evaluation and Management of Chronic Kidney Disease concluded that “there is insufficient evidence to support or refute the use of agents to lower serum UA concentrations to delay progression of CKD” [38]. Some small studies have demonstrated that lowering serum UA levels is beneficial for preventing or slowing kidney disease. Goicoechea et al. reported that allopurinol slowed the progression of CKD [39], and a small randomized study showed that UA lowering therapy improved eGFR among asymptomatic hyperuricemic individuals with normal renal function [40]. In contrast, another larger randomized controlled clinical trial showed no evidence of clinical benefits of serum urate reduction on renal outcomes in patients with type 1 diabetes and early-to-moderate CKD [41]. In addition, in another study of patients with more severe CKD (stage 3 or 4) and a high risk of progression, urate-lowering treatment with allopurinol did not halt the reduction in eGFR [42]. A more recent large cohort study of 269,651 United States veterans without pre-existing CKD reported that UA-lowering therapy was not associated with a lower incidence of CKD, albuminuria or ESRD [43]. Surprisingly, UA-lowering therapy (predominantly allopurinol) was associated with an increased risk of new-onset CKD in the veterans, although the association was limited to those with a baseline serum UA level of ≤8 mg/dL in subgroup analysis [43]. Despite the conflicting clinical trial evidence, UA remains a potential target for avoiding or slowing CKD, thus clinicians should consider urate-lowering agents in patients with marked hyperuricemia and high-risk of developing kidney disease after shared decision-making.

Except for elevated serum UA, we also found other factors in the multiple variable analysis which were significantly correlated with new-onset CKD, including old age, male, the presence of DM or hypertension, higher systolic BP, higher fasting glucose, the habit of alcohol consumption, higher BMI, lower hemoglobin, and higher triglyceride. Age is an independent risk factor for renal disease [44,45] and the pathogenesis of aging-associated global glomerulosclerosis is multifactorial [46]. We also discovered that male patients were at a higher risk of developing kidney disease, which could be explained by estrogen’s protective effect on non-diabetic CKD [47] and other non-biological factors, such as lifestyle, cultural, and socioeconomic factors. Nevertheless, other epidemiological data have shown that the prevalence of CKD is higher in females than in males, which contrasts our findings [48]. Both DM and hypertension are strong cardiovascular risk factor and are also associated with new-onset CKD [48,49]. Furthermore, a large meta-analysis reported an increased risk of developing CKD in prediabetes patient with impaired fasting blood sugar [50]. As for systolic BP, increased systolic BPs were associated with a 35% increased risk of incident CKD for every 10-mm Hg increase in systolic BP according to a large national cohort research [51]. In our study, alcohol consumption was correlated with an increased CKD risk. The role of alcohol consumption as a risk factor for CKD remains controversial. Some population-based studies have shown an association between alcohol consumption and the incidence of CKD [52,53], whereas another study found favorable effect on kidney function [54]. Another modifiable risk factors for developing CKD is being overweight, which may contribute to glomerular hypertrophy and hyperfiltration by increasing capillary wall tension of the glomeruli [55]. A countrywide, population-based study in Sweden discovered that being overweight (BMI ≥ 25 kg/m^2^) at age 20 was related with a considerable threefold increased risk of CKD compared to BMI < 25 kg/m^2^ [56]. Our study revealed that lower hemoglobin increased the risk of CKD development. It is well-known that anemia develops as renal function declines, and correcting anemia is beneficial for improving quality of life, survival, and preventing CKD progression. Nevertheless, limited data show a relationship between low hemoglobin and development of CKD [57]. In addition, we showed the baseline hypertriglyceridemia had a modest effect on new-onset CKD. Similarly, many studies have demonstrated the correlation between hypertriglyceridemia and the initiation of renal insufficiency [58,59].

The strengths of this work include the large number of community-dwelling participants with complete follow-up data of the associations among sex differences with hyperuricemia and new-onset CKD. However, there are also some limitations. First, the TWB does not include information on urate- or lipid-lowering drugs, anti-diabetic drugs, or anti-hypertensive drugs, all of which could have affected the prevention or development of new-onset CKD, serum UA, BP, lipid profiles, and fasting glucose, and consequently the association between hyperuricemia and new-onset CKD. Second, we did not have access to information related to factors which may have caused a rapid worsening of renal function, such as proteinuria. Third, the generalizability of our findings to other groups may be limited as all of the enrolled participants were of Chinese ethnicity. Fourth, sample bias was possible as around a quarter of the participants received follow-up assessments.

## 5. Conclusions

In conclusion, we demonstrated that hyperuricemia was associated with new-onset CKD in this large Taiwanese population follow-up study. Furthermore, the interactions between hyperuricemia and sex were also statistically significant. Hyperuricemia was more strongly associated with new-onset CKD in the women than in the men.

## Figures and Tables

**Table 1:** Comparison of clinical characteristics among participants without or with new-onset CKD.

Characteristics	New-Onset CKD (−) (*n* = 26,360)	New-Onset CKD (+) (*n* = 285)	*p*
Age (year)	51.0 ± 10.4	59.4 ± 6.9	<0.001
Male gender (%)	65.2	38.9	<0.001
DM (%)	4.9	23.2	<0.001
Hypertension (%)	12.3	44.6	<0.001
Systolic BP (mmHg)	117.2 ± 17.5	133.3 ± 19.9	<0.001
Diastolic BP (mmHg)	72.4 ± 10.8	77.8 ± 11.6	<0.001
Smoking history (%)	25.3	41.8	<0.001
Alcohol history (%)	2.8	8.4	<0.001
Regular exercise habits (%)	48.1	51.9	0.203
BMI (kg/m^2^)	24.0 ± 3.5	26.1 ± 4.0	<0.001
Menstruation in female (%)	45.3	14.4	<0.001
Laboratory parameters			
Uric acid (mg/dL)	5.4 ± 1.4	6.7 ± 1.5	<0.001
Hyperuricemia (%)	19.7	48.4	<0.001
Fasting glucose (mg/dL)	95.9 ± 19.5	113.3 ± 42.3	<0.001
Hemoglobin (g/dL)	13.7 ± 1.5	14.0 ± 1.6	0.002
Triglyceride (mg/dL)	113.1 ± 82.2	161.5 ± 127.5	<0.001
Total cholesterol (mg/dL)	195.4 ± 35.3	196.3 ± 41.4	0.658
HDL-cholesterol (mg/dL)	54.4 ± 13.2	48.5 ± 12.4	<0.001
LDL-cholesterol (mg/dL)	121.7 ± 31.6	119.9 ± 33.5	0.350
eGFR (mL/min/1.73 m^2^)	115.2 ± 24.5	76.6 ± 16.1	<0.001

Abbreviations: DM, diabetes mellitus; BP, blood pressure; BMI, body mass index; HDL, high-density lipoprotein; LDL, low-density lipoprotein; eGFR, estimated glomerular filtration rate. Hyperuricemia is defined as participants with serum uric acid >7 mg/dL in male and >6 mg/dL in female.

**Table 2:** Determinants for new-onset CKD using multivariable logistic regression analysis.

Parameters	New-Onset CKD		
	Multivariable		
	OR	95% CI	*p*
Age (per 1 year)	1.079	1.060–1.098	<0.001
Female (vs. male)	0.340	0.239–0.484	<0.001
DM	1.676	1.183–2.375	0.004
Hypertension	1.740	1.327–2.281	<0.001
Systolic BP (per 1 mmHg)	1.021	1.012–1.031	<0.001
Diastolic BP (per 1 mmHg)	0.996	0.980–1.012	0.595
Smoking history	1.095	0.804–1.491	0.564
Alcohol history	2.081	1.308–3.311	0.002
BMI (per 1 kg/m^2^)	1.065	1.027–1.103	0.001
Laboratory parameters			
Hyperuricemia	2.541	1.970–3.276	<0.001
Fasting glucose (per 1 mg/dL)	1.009	1.005–1.013	<0.001
Hemoglobin (per 1 g/dL)	0.762	0.692–0.89	<0.001
Triglyceride (per 1 mg/dL)	1.002	1.001–1.003	<0.001
HDL-cholesterol (per 1 mg/dL)	1.002	0.990–1.014	0.743

Values expressed as odds ratio (OR) and 95% confidence interval (CI). Abbreviations are the same as in Table 1. Hyperuricemia is defined as participants with serum uric acid >7 mg/dL in male and >6 mg/dL in female. Adjusted for age, sex, diabetes, hypertension, systolic and diastolic blood pressures, smoking and alcohol history, body mass index, fasting glucose, hemoglobin, triglyceride and HDL-cholesterol.

**Table 3:** Clinical characteristics of the study participants classified by the presence of different sex and hyperuricemia.

Characteristics	Male (*n* = 9356)			Female (*n* = 17,289)		
	Hyperuricemia (−) (*n* = 6532)	Hyperuricemia (+) (*n* = 2824)	*p*	Hyperuricemia (−) (*n* = 14,773)	Hyperuricemia (+) (*n* = 2516)	*p*
Age (year)	51.9 ± 10.8	49.9 ± 11.0	<0.001	50.5 ± 10.1	54.2 ± 9.3	<0.001
DM (%)	7.5	4.7	<0.001	3.6	7.8	<0.001
Hypertension (%)	15.7	19.9	<0.001	8.3	22.3	<0.001
Systolic BP (mmHg)	121.6 ± 16.5	123.4 ± 15.7	<0.001	113.6 ± 17.3	122.6 ± 18.2	<0.001
Diastolic BP (mmHg)	76.2 ± 10.4	78.8 ± 10.2	<0.001	69.3 ± 10.1	74.0 ± 10.4	<0.001
Smoking history (%)	57.3	62.4	<0.001	7.4	7.6	0.637
Alcohol history (%)	6.3	8.9	<0.001	0.6	0.9	0.125
Regular exercise habits (%)	50.1	46.7	0.003	47.7	47.9	0.846
BMI (kg/m^2^)	24.5 ± 3.1	26.3 ± 3.4	<0.001	23.1 ± 3.3	26.1 ± 4.1	<0.001
Menstruation in female (%)	–	–		47.8	29.0	<0.001
Laboratory parameters						
Uric acid (mg/dL)	5.8 ± 0.9	8.0 ± 0.8	<0.001	4.6 ± 0.8	6.8 ± 0.8	<0.001
Fasting glucose (mg/dL)	100.2 ± 24.7	95.4 ± 15.6	<0.001	93.4 ± 17.9	98.5 ± 19.1	<0.001
Hemoglobin (g/dL)	15.0 ± 1.2	15.2 ± 1.1	<0.001	13.0 ± 1.3	13.4 ± 1.1	<0.001
Triglyceride (mg/dL)	121.1 ± 89.5	161.4 ± 117.5	<0.001	96.8 ± 65.3	138.9 ± 80.5	<0.001
Total cholesterol (mg/dL)	189.6 ± 33.7	195.9 ± 357.8	<0.001	196.0 ± 35.2	206.3 ± 37.2	<0.001
HDL-cholesterol (mg/dL)	49.1 ± 11.2	45.6 ± 10.1	<0.001	58.7 ± 13.0	52.1 ± 11.6	<0.001
LDL-cholesterol (mg/dL)	121.0 ± 30.7	124.7 ± 32.6	<0.001	119.8 ± 31.1	130.8 ± 33.5	<0.001
eGFR (mL/min/1.73 m^2^)	102.7 ± 19.8	93.9 ± 18.0	<0.001	117.2 ± 25.3	103.1 ± 22.7	<0.001

Abbreviations are the same as in Table 1.

**Table 4:** Association of hyperuricemia and quartiles of uric acid with new-onset CKD using multivariable logistic regression analysis in different sex.

Parameters	Male (*n* = 9356)			Female (*n* = 17,289)			
	Multivariable *			Multivariable ^#^			
	OR	95% CI	*p*	OR	95% CI	*p*	Interaction *p*
Hyperuricemia	1.989	1.440–2.747	<0.001	3.813	2.500–5.815	<0.001	0.024
Serum uric acid							
Quartile 1	Reference			Reference			0.010
Quartile 2	0.998	0.616–1.618	0.994	2.577	0.810–8.196	0.109	
Quartile 3	1.122	0.685–1.833	0.647	3.741	1.250–11.915	0.018	
Quartile 4	2.279	1.464–3.547	<0.001	12.114	4.278–34.305	<0.001	

Values expressed as odds ratio (OR) and 95% confidence interval (CI). Hyperuricemia is defined as participants with serum uric acid >7 mg/dL in male and >6 mg/dL in female. The cut-off values of quartiles of serum uric acid were ≤5.5, 5.5–6.4, 6.4–7.3 and >7.3 mg/dL in male participants, and ≤4.1, 4.1–4.8, 4.8–5.6 and >5.6 mg/dL in female participants. * Adjusted for age, diabetes, hypertension, systolic and diastolic blood pressures, smoking and alcohol history, body mass index, fasting glucose, hemoglobin, triglyceride and HDL-cholesterol. ^#^ Adjusted for age, diabetes, hypertension, systolic and diastolic blood pressures, smoking and alcohol history, body mass index, fasting glucose, hemoglobin, triglyceride, HDL-cholesterol and menopause status.

## Data Availability

The data underlying this study are from the Taiwan Biobank. Due to restrictions placed on the data by the Personal Information Protection Act of Taiwan, the minimal data set cannot be made publicly available. Data may be available upon request to interested researchers. Please send data requests to: Szu-Chia Chen, PhD, MD. Division of Nephrology, Department of Internal Medicine, Kaohsiung Medical University Hospital, Kaohsiung Medical University.

## References

[1] Lai T.S., Hsu C.C., Lin M.H., Wu V.C., Chen Y.M. (2022). Trends in the incidence and prevalence of end-stage kidney disease requiring dialysis in Taiwan: 2010–2018. J. Formos. Med. Assoc..

[2] Yamagata K., Yagisawa T., Nakai S., Nakayama M., Imai E., Hattori M., Iseki K., Akiba T. (2015). Prevalence and incidence of chronic kidney disease stage G5 in Japan. Clin. Exp. Nephrol..

[3] Jager K.J., Kovesdy C., Langham R., Rosenberg M., Jha V., Zoccali C. (2019). A single number for advocacy and communication-worldwide more than 850 million individuals have kidney diseases. Kidney Int..

[4] Nelson A.J., Raggi P., Wolf M., Gold A.M., Chertow G.M., Roe M.T. (2020). Targeting Vascular Calcification in Chronic Kidney Disease. JACC Basic Transl. Sci..

[5] Chen T.K., Knicely D.H., Grams M.E. (2019). Chronic Kidney Disease Diagnosis and Management: A Review. JAMA.

[6] Benn C.L., Dua P., Gurrell R., Loudon P., Pike A., Storer R.I., Vangjeli C. (2018). Physiology of Hyperuricemia and Urate-Lowering Treatments. Front. Med..

[7] Feig D.I., Kang D.H., Johnson R.J. (2008). Uric acid and cardiovascular risk. N. Engl. J. Med..

[8] Ben-Dov Z.I., Kark J.D. (2011). Serum uric acid is a GFR-independent long-term predictor of acute and chronic renal insufficiency: The Jerusalem Lipid Research Clinic cohort study. Nephrol. Dial. Transplant..

[9] Iseki K., Oshiro S., Tozawa M., Iseki C., Ikemiya Y., Takishita S. (2001). Significance of hyperuricemia on the early detection of renal failure in a cohort of screened subjects. Hypertens. Res..

[10] Obermayr R.P., Temml C., Gutjahr G., Knechtelsdorfer M., Oberbauer R., Klauser-Braun R. (2008). Elevated uric acid increases the risk for kidney disease. J. Am. Soc. Nephrol..

[11] Weiner D.E., Tighiouart H., Elsayed E.F., Griffith J.L., Salem D.N., Levey A.S. (2008). Uric acid and incident kidney disease in the community. J. Am. Soc. Nephrol..

[12] Liu W.C., Hung C.C., Chen S.C., Yeh S.M., Lin M.Y., Chiu Y.W., Kuo M.C., Chang J.M., Hwang S.J., Chen H.C. (2012). Association of hyperuricemia with renal outcomes, cardiovascular disease, and mortality. Clin. J. Am. Soc. Nephrol..

[13] Adamopoulos D., Vlassopoulos C., Seitanides B., Contoyiannis P., Vassilopoulos P. (1977). The relationship of sex steroids to uric acid levels in plasma and urine. Acta Endocrinol..

[14] Chen C.H., Yang J.H., Chiang C.W.K., Hsiung C.N., Wu P.E., Chang L.C., Chu H.W., Chang J., Song I.W., Yang S.L. (2016). Population structure of Han Chinese in the modern Taiwanese population based on 10,000 participants in the Taiwan Biobank project. Hum. Mol. Genet..

[15] Fan C.T., Hung T.H., Yeh C.K. (2015). Taiwan Regulation of Biobanks. J. Law Med. Ethics.

[16] Levey A.S., Bosch J.P., Lewis J.B., Greene T., Rogers N., Roth D. (1999). A more accurate method to estimate glomerular filtration rate from serum creatinine: A new prediction equation. Modification of Diet in Renal Disease Study Group. Ann. Intern. Med..

[17] Isomaa B., Henricsson M., Almgren P., Tuomi T., Taskinen M.R., Groop L. (2001). The metabolic syndrome influences the risk of chronic complications in patients with type II diabetes. Diabetologia.

[18] Lee J.W., Kwon B.C., Choi H.G. (2021). Analyses of the relationship between hyperuricemia and osteoporosis. Sci. Rep..

[19] Chonchol M., Shlipak M.G., Katz R., Sarnak M.J., Newman A.B., Siscovick D.S., Kestenbaum B., Carney J.K., Fried L.F. (2007). Relationship of uric acid with progression of kidney disease. Am. J. Kidney Dis..

[20] Mazzali M., Kanellis J., Han L., Feng L., Xia Y.Y., Chen Q., Kang D.H., Gordon K.L., Watanabe S., Nakagawa T. (2002). Hyperuricemia induces a primary renal arteriolopathy in rats by a blood pressure-independent mechanism. Am. J. Physiol. Renal. Physiol..

[21] Sanchez-Lozada L.G., Tapia E., Santamaria J., Avila-Casado C., Soto V., Nepomuceno T., Rodriguez-Iturbe B., Johnson R.J., Herrera-Acosta J. (2005). Mild hyperuricemia induces vasoconstriction and maintains glomerular hypertension in normal and remnant kidney rats. Kidney Int..

[22] Corry D.B., Eslami P., Yamamoto K., Nyby M.D., Makino H., Tuck M.L. (2008). Uric acid stimulates vascular smooth muscle cell proliferation and oxidative stress via the vascular renin-angiotensin system. J. Hypertens..

[23] Sanchez-Lozada L.G., Soto V., Tapia E., Avila-Casado C., Sautin Y.Y., Nakagawa T., Franco M., Rodriguez-Iturbe B., Johnson R.J. (2008). Role of oxidative stress in the renal abnormalities induced by experimental hyperuricemia. Am. J. Physiol. Renal. Physiol..

[24] Ryu E.S., Kim M.J., Shin H.S., Jang Y.H., Choi H.S., Jo I., Johnson R.J., Kang D.H. (2013). Uric acid-induced phenotypic transition of renal tubular cells as a novel mechanism of chronic kidney disease. Am. J. Physiol. Renal. Physiol..

[25] Sellmayr M., Hernandez Petzsche M.R., Ma Q., Kruger N., Liapis H., Brink A., Lenz B., Angelotti M.L., Gnemmi V., Kuppe C. (2020). Only Hyperuricemia with Crystalluria, but not Asymptomatic Hyperuricemia, Drives Progression of Chronic Kidney Disease. J. Am. Soc. Nephrol..

[26] Kuwabara M., Niwa K., Nishi Y., Mizuno A., Asano T., Masuda K., Komatsu I., Yamazoe M., Takahashi O., Hisatome I. (2014). Relationship between serum uric acid levels and hypertension among Japanese individuals not treated for hyperuricemia and hypertension. Hypertens. Res..

[27] Forman J.P., Choi H., Curhan G.C. (2007). Plasma uric acid level and risk for incident hypertension among men. J. Am. Soc. Nephrol..

[28] Mellen P.B., Bleyer A.J., Erlinger T.P., Evans G.W., Nieto F.J., Wagenknecht L.E., Wofford M.R., Herrington D.M. (2006). Serum uric acid predicts incident hypertension in a biethnic cohort: The atherosclerosis risk in communities study. Hypertension.

[29] Lehto S., Niskanen L., Ronnemaa T., Laakso M. (1998). Serum uric acid is a strong predictor of stroke in patients with non-insulin-dependent diabetes mellitus. Stroke.

[30] Dehghan A., van Hoek M., Sijbrands E.J., Hofman A., Witteman J.C. (2008). High serum uric acid as a novel risk factor for type 2 diabetes. Diabetes Care.

[31] Cicero A.F., Salvi P., D'Addato S., Rosticci M., Borghi C., Brisighella Heart Study group (2014). Association between serum uric acid, hypertension, vascular stiffness and subclinical atherosclerosis: Data from the Brisighella Heart Study. J. Hypertens..

[32] Mallamaci F., Testa A., Leonardis D., Tripepi R., Pisano A., Spoto B., Sanguedolce M.C., Parlongo R.M., Tripepi G., Zoccali C. (2015). A genetic marker of uric acid level, carotid atherosclerosis, and arterial stiffness: A family-based study. Am. J. Kidney Dis..

[33] Li L., Yang C., Zhao Y., Zeng X., Liu F., Fu P. (2014). Is hyperuricemia an independent risk factor for new-onset chronic kidney disease?: A systematic review and meta-analysis based on observational cohort studies. BMC Nephrol..

[34] Nakayama S., Satoh M., Tatsumi Y., Murakami T., Muroya T., Hirose T., Ohkubo T., Mori T., Hozawa A., Metoki H. (2021). Detailed association between serum uric acid levels and the incidence of chronic kidney disease stratified by sex in middle-aged adults. Atherosclerosis.

[35] Yang Y., Zhou W., Wang Y., Zhou R. (2019). Gender-specific association between uric acid level and chronic kidney disease in the elderly health checkup population in China. Ren. Fail..

[36] Wang Y., Charchar F.J. (2021). Establishment of sex difference in circulating uric acid is associated with higher testosterone and lower sex hormone-binding globulin in adolescent boys. Sci. Rep..

[37] Huh K., Shin U.S., Choi J.W., Lee S.I. (1994). Effect of sex hormones on lipid peroxidation in rat liver. Arch Pharm. Res..

[38] Stevens P.E., Levin A., Kidney M. (2013). Disease: Improving Global Outcomes Chronic Kidney Disease Guideline Development Work Group, Evaluation and management of chronic kidney disease: Synopsis of the kidney disease: Improving global outcomes 2012 clinical practice guideline. Ann. Intern. Med..

[39] Goicoechea M., de Vinuesa S.G., Verdalles U., Ruiz-Caro C., Ampuero J., Rincon A., Arroyo D., Luno J. (2010). Effect of allopurinol in chronic kidney disease progression and cardiovascular risk. Clin. J. Am. Soc. Nephrol..

[40] Kanbay M., Huddam B., Azak A., Solak Y., Kadioglu G.K., Kirbas I., Duranay M., Covic A., Johnson R.J. (2011). A randomized study of allopurinol on endothelial function and estimated glomular filtration rate in asymptomatic hyperuricemic subjects with normal renal function. Clin. J. Am. Soc. Nephrol..

[41] Doria A., Galecki A.T., Spino C., Pop-Busui R., Cherney D.Z., Lingvay I., Parsa A., Rossing P., Sigal R.J., Afkarian M. (2020). Serum Urate Lowering with Allopurinol and Kidney Function in Type 1 Diabetes. N. Engl. J. Med..

[42] Badve S.V., Pascoe E.M., Tiku A., Boudville N., Brown F.G., Cass A., Clarke P., Dalbeth N., Day R.O., de Zoysa J.R. (2020). Effects of Allopurinol on the Progression of Chronic Kidney Disease. N. Engl. J. Med..

[43] Hassan W., Shrestha P., Sumida K., Thomas F., Sweeney P.L., Potukuchi P.K., Rhee C.M., Streja E., Kalantar-Zadeh K., Kovesdy C.P. (2022). Association of Uric Acid-Lowering Therapy With Incident Chronic Kidney Disease. JAMA Netw. Open.

[44] Fox C.S., Larson M.G., Leip E.P., Culleton B., Wilson P.W., Levy D. (2004). Predictors of new-onset kidney diseas.se in a community-based population. JAMA.

[45] Yamagata K., Ishida K., Sairenchi T., Takahashi H., Ohba S., Shiigai T., Narita M., Koyama A. (2007). Risk factors for chronic kidney disease in a community-based population: A 10-year follow-up study. Kidney Int..

[46] Zhou X.J., Rakheja D., Yu X., Saxena R., Vaziri N.D., Silva F.G. (2008). The aging kidney. Kidney Int..

[47] Silbiger S.R., Neugarten J. (1995). The impact of gender on the progression of chronic renal disease. Am. J. Kidney Dis..

[48] Kovesdy C.P. (2022). Epidemiology of chronic kidney disease: An update 2022. Kidney Int. Suppl..

[49] Kazancioglu R. (2013). Risk factors for chronic kidney disease: An update. Kidney Int. Suppl..

[50] Echouffo-Tcheugui J.B., Narayan K.M., Weisman D., Golden S.H., Jaar B.G. (2016). Association between prediabetes and risk of chronic kidney disease: A systematic review and meta-analysis. Diabet Med..

[51] Chang T.I., Lim H., Park C.H., Rhee C.M., Moradi H., Kalantar-Zadeh K., Kang E.W., Kang S.W., Han S.H. (2020). Associations of Systolic Blood Pressure With Incident CKD G3-G5: A Cohort Study of South Korean Adults. Am. J. Kidney Dis..

[52] Lai Y.J., Chen Y.Y., Lin Y.K., Chen C.C., Yen Y.F., Deng C.Y. (2019). Alcohol Consumption and Risk of Chronic Kidney Disease: A Nationwide Observational Cohort Study. Nutrients.

[53] Tanaka A., Yamaguchi M., Ishimoto T., Katsuno T., Nobata H., Iwagaitsu S., Sugiyama H., Kinashi H., Banno S., Imaizumi T. (2022). Association of alcohol consumption with the incidence of proteinuria and chronic kidney disease: A retrospective cohort study in Japan. Nutr. J..

[54] Lee Y.J., Cho S., Kim S.R. (2021). Effect of alcohol consumption on kidney function: Population-based cohort study. Sci. Rep..

[55] Chang A., Kramer H. (2012). CKD progression: A risky business. Nephrol. Dial. Transplant..

[56] Ejerblad E., Fored C.M., Lindblad P., Fryzek J., McLaughlin J.K., Nyren O. (2006). Obesity and risk for chronic renal failure. J. Am. Soc. Nephrol..

[57] Iseki K., Kohagura K. (2007). Anemia as a risk factor for chronic kidney disease. Kidney Int. Suppl..

[58] Muntner P., Coresh J., Smith J.C., Eckfeldt J., Klag M.J. (2000). Plasma lipids and risk of developing renal dysfunction: The atherosclerosis risk in communities study. Kidney Int..

[59] Hadjadj S., Duly-Bouhanick B., Bekherraz A., BrIdoux F., Gallois Y., Mauco G., Ebran J., Marre M. (2004). Serum triglycerides are a predictive factor for the development and the progression of renal and retinal complications in patients with type 1 diabetes. Diabetes Metab..

